# Clinical guidelines for the treatment of perinatal depressive disorder

**DOI:** 10.1192/j.eurpsy.2026.11395

**Published:** 2026-07-31

**Authors:** J. V. Stojanov

**Affiliations:** Centre for mental health protection Clinical Centre Nis, Nis, Serbia

## Abstract

**Introduction:**

Perinatal depressive disorder (PPD) is a common depressive mood disorder that occurs during pregnancy or within a year after childbirth with a prevalence of 25% in our country and with the most frequent occurrence in the first six months after childbirth (1).

**Objectives:**

Untreated or inadequately treated PPD is associated with an increased risk of future depressive episodes, abuse psychoactive substances, problems in partner relationships and suicide, along with far-reaching consequences on offspring (2, 3).

**Methods:**

Despite the significant frequency of PPD, diagnosis and treatment are not up to date satisfactory level, which is due to insufficient information of the general and expert public, as well as due to lack of precise guidance guidelines narrowly specific for this vulnerable population.

**Results:**

The latest a Canadian network guideline for the treatment of a wide range of perinatal disorders, including PPD (CANMAT 2024) aims to provide detailed evidence-based comprehensive and systematized guidelines that provide clinicians with key information to promote provision effective and safe perinatal mental health care (3).

**Conclusions:**

The lecture will show recommendations for identification of PPD, organization and provision of perinatal mental services health care, the use of psychosocial, psychological, pharmacological, neuromodulation, complementary and alternative interventions in the prevention and treatment of PPD, as well as recommendations for management of high-risk clinical situations with reference to the mental health of the father or other parents and additional considerations on equality, diversity and inclusion.

**Disclosure of Interest:**

None Declared

